# Emerging PET Imaging Agents and Targeted Radioligand Therapy: A Review of Clinical Applications and Trials

**DOI:** 10.3390/tomography11080083

**Published:** 2025-07-28

**Authors:** Maierdan Palihati, Jeeban Paul Das, Randy Yeh, Kathleen Capaccione

**Affiliations:** 1 Department of Radiology, Columbia University Irving Medical Center, 622 W 168th St., New York, NY 10032, USA; 2 Department of Radiology, Memorial Sloan Kettering Cancer Center, New York, NY 10065, USA

**Keywords:** radiopharmaceuticals, fibroblast activation protein (FAP), hypoxia, gastrin-releasing peptide receptors (GRPrs), integrins, clinical trials

## Abstract

Targeted radioligand therapy (RLT) is an emerging field in anticancer therapeutics with great potential across tumor types and stages of disease. While much progress has focused on agents targeting somatostatin receptors and prostate-specific membrane antigen (PSMA), the same advanced radioconjugation methods and molecular targeting have spurred the development of numerous theranostic combinations for other targets. A number of the most promising agents have progressed to clinical trials and are poised to change the landscape of positron emission tomography (PET) imaging. Here, we present recent data on some of the most important emerging molecular targeted agents with their exemplar clinical images, including agents targeting fibroblast activation protein (FAP), hypoxia markers, gastrin-releasing peptide receptors (GRPrs), and integrins. These radiopharmaceuticals share the promising characteristic of being able to image multiple types of cancer. Early clinical trials have already demonstrated superiority to ^18^F-fluorodeoxyglucose (^18^F-FDG) for some, suggesting the potential to supplant this longstanding PET radiotracer. Here, we provide a primer for practicing radiologists, particularly nuclear medicine clinicians, to understand novel PET imaging agents and their clinical applications, as well as the availability of companion targeted radiotherapeutics, the status of their regulatory approval, the potential challenges associated with their use, and the future opportunities and perspectives.

## 1. Introduction

The use of radiation for imaging and therapy dates back more than one hundred years. Historically, a limited but highly effective set of theranostic imaging and/or therapy agents had been approved in the United States, including ^123^I/^131^I for hyperthyroidism [1], ^90^Y-ibritumomab tiuxetan (Zevalin) for refractory B-cell non-Hodgkin’s lymphoma [2], ^131^I-metaiodobenzylguanidine (^131^I-MIBG) for pheochromocytoma [3], ^153^Samarium-ethylene diamine tetramethylene phosphonate (^153^Samarium-EDTMP) [4,5], and ^89^Strontium [6] for palliation of bone metastasis.

More recently, theranostic imaging and therapy pairs for somatostatin receptor (SSTR)-positive neuroendocrine tumors demonstrated increased progression-free survival and response rates [7]. Theranostic agents for prostate cancer targeting prostate-specific membrane antigen (PSMA) have had similar success and have been reviewed elsewhere [8]. Here, we describe emerging pan-cancer PET imaging agents that are likely to significantly change the face of cancer imaging, together with the status of their regulatory approval, the potential challenges associated with their use, and the future opportunities and perspectives.

## 2. Emerging PET Imaging Agents and Combination Radiotherapies

**Figure 1:**
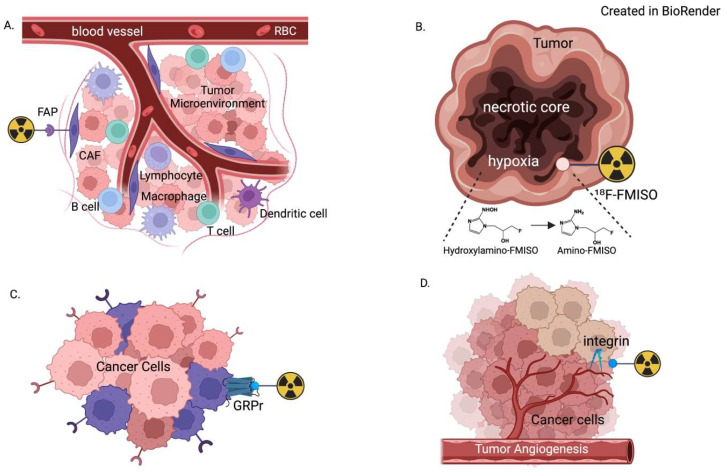
Demonstration of the molecular context of radioligands targeting (**A**) fibroblast activation protein (FAP); (**B**) hypoxia; (**C**) gastrin-releasing peptide receptor (GRPr); and (**D**) integrin. Each is part of the complex tumor microenvironment, which allows for high tumor-to-background uptake ratios on PET imaging.

Given that progress in chemistry and molecular biology has improved the specificity and tumor retention time of ligands, numerous agents have been developed for tumor targeting. They broadly fall into categories of pan-cancer versus disease-specific ligands. Here, we detail exciting advances of leading pan-cancer theranostic agents targeting fibroblast activation protein (FAP), hypoxia, gastrin-releasing peptide receptors (GRPrs), and integrins, respectively, which offer the promise of significantly impacting cancer imaging and therapy in the coming years. To investigate these agents, we conducted a targeted literature review to identify key clinical trials and publications. Our process for literature identification, detailing the databases searched, as well as the inclusion and exclusion criteria applied during the screening and eligibility assessment of trials and publications, is included in Supplementary Figure S1. Figure 1 is a schematic overview of each of the agents targeting (A) fibroblast activation protein (FAP); (B) hypoxia; (C) gastrin-releasing peptide receptors (GRPrs); and (D) integrins; Table 1, Table 2, Table 3 and Table 4 summarize ongoing clinical trials for these agents, respectively.

### 2.1. Fibroblast Activation Protein Targeting Agents

**Figure 2:**
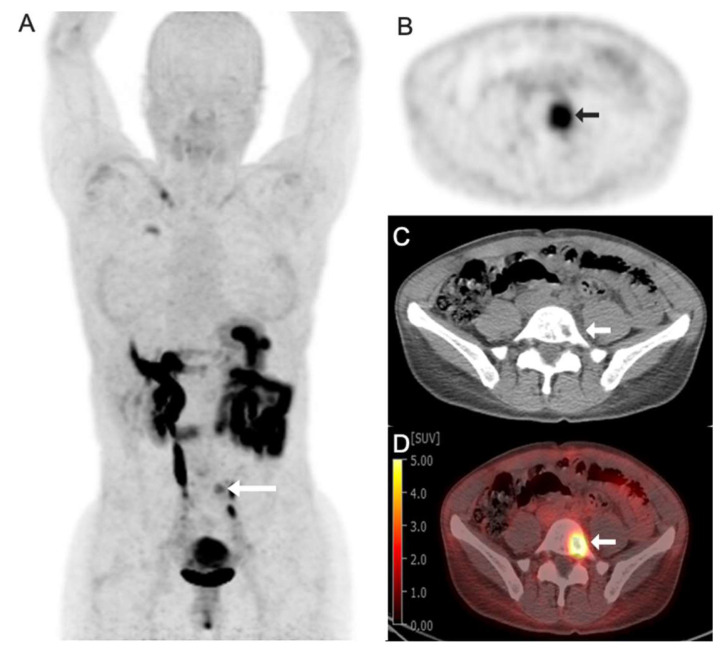
Images obtained from a 42-year-old woman with gastric cancer with focal radiotracer uptake in left L5 vertebral body lytic metastasis (arrows). (**A**) Fluorine 18 (^18^F)-labeled fibroblast activation protein inhibitor-74 (FAPI-74) maximum intensity projection image (MIP); (**B**) axial PET image; (**C**) axial CT image; (**D**) axial fused ^18^F-FAPI-74 PET/CT image with a Standard Uptake Value (SUV) max of 7.0.

Fibroblast activation protein (FAP) is a serine protease that is present in activated fibroblasts in cancer and other disease states, such as pulmonary fibrosis. In tumor stroma, these activated fibroblasts are termed cancer-associated fibroblasts (CAFs) and are important contributors to the tumor microenvironment [86]. Because these cells are ubiquitous in both primary and metastatic cancer types, particularly across solid tumors, it is an attractive pan-tumor target. Early chemistry studies performed led to optimization on a quinoline-based molecule, resulting in the “FAPI” (FAP Inhibitor) series of molecules, which were early leaders in targeting FAP for PET imaging and therapy [87]. Preclinical models demonstrated excellent tumor-to-background ratios using these molecules for PET imaging and exciting efficacy using these molecules to target a radioactive payload to tumors [88]. This success led to clinical translation in conjunction with further refinements in the molecular structure of FAPI molecules to increase tumor retention time and increase their versatility. Figure 2 shows example 18F-FAPI-74 clinical images obtained from a 42-year-old woman with gastric cancer. An international retrospective multicenter analysis included PET/CT data from 71 patients who underwent both 68Ga-FAPI and 18F-FDG PET/CT concluded that quantitative tumor uptake is comparable between ^68^Ga-FAPI and ^18^F-FDG, but lower background uptake in most normal organs resulted in equal or higher tumor-to-background ratios for ^68^Ga-FAPI, such that ^68^Ga-FAPI PET/CT may yield improved diagnostic information in various cancers and especially in tumor locations with high physiological ^18^F-FDG uptake [89]. A study using ^68^Ga-FAPI-04 evaluating PET/CT imaging in 28 different types of cancer demonstrated excellent tumor-to-background ratios in sarcoma, esophageal, breast, cholangiocarcinoma, lung cancer, hepatocellular, colorectal, head–neck, ovarian, pancreatic, and prostate cancer, some of which are traditionally poorly visualized on ^18^F-FDG PET [90]. Mori et al. recently reviewed a head-to-head comparison and presented representative images between ^18^F-FDG and ^18^F-FAPI-74 PET imaging, ^18^F-FDG and ^68^Ga-FAPI-04 PET imaging in oncologic patients with various cancer types, and demonstrated the advantages of FAP targeting imaging agents [91]. The authors also concluded that miscellaneous studies comparing sensitivity of FAPI-PET to FDG-PET in hepatocellular carcinomas (96 to 100% vs. 50 to 80%), primary cholangiocarcinoma (98% vs. 86%) and metastases, pancreatic ductal adenocarcinoma (100% vs. 95%), gastric cancers, primary lung cancer and metastases, primary colorectal cancer (100% vs. 53%) and metastases, primary breast cancers and metastases, and peritoneal carcinomatosis, have documented superiority of FAPI-PET in sensitivity. However, they pointed out that lower specificity in staging lung cancer is a limitation of FAPI-PET due to false-positive uptake of various benign conditions (co-existing post-radiation injury, surgery, or inflammation).

In parallel, other FAP targeting molecules with different molecular structures were developed and tested. A recent systematic review and meta-analysis of FAP PET imaging agents assessed 30 studies with 1170 patients and found a diagnostic odds ratio (used for estimation of discriminative power of diagnostic procedures and the comparison of diagnostic accuracies between diagnostic tests, defined as the ratio of the odds of positivity in subjects with disease relative to the odds in subjects without disease [92]) of 19.38–358.47 compared to ^18^F-FDG and concluded that FAP based radiotracers could replace FDG PET for most applications due to their improved sensitively, with the exception of urological system cancer [93]. Currently, several are in various stages of clinical trials, and, as these trials read out, it will become clear which FAP imaging agents will have the greatest impact on clinical imaging.

FAPI-46 is a descendant of the early FAPI molecules and has shown dramatic success in clinical trials. Ongoing trials of FAPI-46 include studies of breast cancer (ER-positive, lobular, and triple-negative cancer) [9], lung cancer [10], pancreatic/bile duct cancers [13], hepatocellular carcinoma, cancer of unknown primary [17], prostate cancer, sarcoma [19], and ovarian cancers [15], as well as trials enrolling multiple tumor types. In addition to validation of this tracer in multiple tumor types in large cohorts, other studies are pressing the boundaries of PET imaging. One ongoing study in early-stage patients with triple-negative breast cancer is studying the potential to use ^68^Ga-FAP PET imaging to predict histological response [22]. A similar study in patients with ovarian cancer seeks to identify recurrent disease in patients who had achieved a complete response [23]. Findings from these studies may expand the utility of FAP PET imaging and provide new clinical tools for oncology. FAP-2286 is the leading FAP targeting agent that did not emerge from the FAPI series of molecules, it differs from FAPI molecules primarily in its binding motif to target FAP, a cyclic peptide structure instead of the quinoline-based structure used by FAPI molecules. The study of its initial validation and biodistribution was published in *the European Journal of Nuclear Medicine and Molecular Imaging* in 2022 [94], and it is predominantly used as a combination theranostic with ^177^Lu-FAP-2286. The LuMIERE phase 1/2 study reported the tolerability, pharmacokinetics, dosimetry, and preliminary activity of the radiotherapy in patients with positive FAP-2286 PET uptake [95]. To date, 48 patients with breast, bladder, prostate, colon, head/neck, sarcoma, cholangiocarcinoma, and lung cancer have been treated. While the full results will not be read out until after the study conclusion in 2026, early data has shown excellent lesion characterization with ^68^Ga-FAP-2286 PET imaging and anticancer efficacy of ^177^Lu-FAP-2286 against tumors. This theranostic combination is promising and may become an important option, especially in tumors with few treatment options. Recently, a group performed a comparative study of ^68^Ga-FAPI-46, ^68^Ga-FAP-2286, and ^18^F-FDG PET. They found that both of the FAP imaging agents had comparable lesion detection and demonstrated superior lesion detection compared to FDG PET [96]. Additional compounds targeting FAP are in various stages of development [97], and a FAP agent may become the standard of care for cancer imaging. However, the widespread clinical adoption of FAP-targeting tracers is currently limited by supply constraints and high production costs, which remain prohibitive in many regions. Indeed, these concerns may be true for all radiopharmaceutical agents, given the challenges of supplying radionuclides and the specialized expertise needed for on-site production of certain agents. Addressing these challenges will require increased radiopharmaceutical production capacity, streamlined regulatory approvals, and cost-reduction strategies such as centralized manufacturing and broader distribution networks.

### 2.2. Hypoxia Imaging Agents

**Figure 3:**
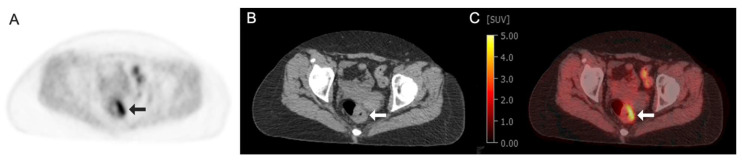
Images obtained from a 68-year-old man with rectal cancer demonstrating focal circumferential radiotracer uptake in left rectal wall thickening (arrows) at the site of primary tumor: (**A**) Fluorine 18 (^18^F)-labeled Fluoromisonidazole (FMISO) axial PET image; (**B**) axial CT image; (**C**) axial fused ^18^F-FMISO PET/CT image with an SUV max of 5.4.

**Figure 4:**
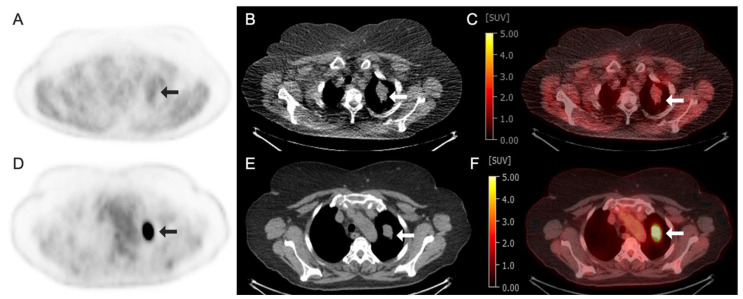
Images obtained from a 70-year-old woman with non-small-cell lung cancer demonstrating radiotracer-avid primary lung neoplasm at the left lung apex (arrows). (**A**–**C**) Fluorine 18 (^18^F)-labeled Fluoromisonidazole (FMISO) PET/CT images. (**A**) ^18^F–FMISO axial PET image; (**B**) axial CT image; (**C**) axial fused ^18^F-FMISO PET/CT image with an SUV max of 2.5. (**D**–**F**) Fluorine 18 (^18^F)-labeled Fluorodeoxyglucose (FDG) PET/CT images of the same patient performed 1 week prior. (**D**) ^18^F-FDG axial PET image; (**E**) axial CT image; (**F**) axial fused ^18^F-FDG PET/CT image demonstrating intense avidity with an SUV max of 24.

^18^F-Fluoromisonidazole (^18^F-FMISO) is a radiotracer widely used to detect hypoxia, a hallmark of many tumors and certain ischemic diseases. Hypoxia promotes angiogenesis, cancer invasiveness, and resistance to therapy [98]. ^18^F-FMISO is a nitroimidazole compound that selectively accumulates in hypoxic tissues due to its bioreductive properties, undergoing intracellular trapping under low-oxygen conditions and irreversible binding within hypoxic regions. The unique hypoxia-targeting properties of ^18^F-FMISO have made it an important tool for both research and clinical oncology, helping clinicians assess tumor oxygenation and guide treatment planning. Figure 3 and Figure 4 show the example ^18^F-FMISO clinical images obtained from a 68-year-old man with rectal cancer and a 70-year-old woman with non-small-cell lung cancer, respectively.

Preclinical studies demonstrated that ^18^F-FMISO could achieve a high contrast between hypoxic and well-oxygenated tissues, showing a strong correlation between tracer uptake and hypoxic markers in cancer models. In preclinical models of breast and colon cancer, the tumor and its surrounding microenvironment were probed with ^18^F-FMISO PET imaging to noninvasively quantify hypoxia in vivo prior to and during PD-1 and CTLA-4 immune checkpoint blockade [99]. In recent years, clinical trials have assessed ^18^F-FMISO PET imaging of hypoxia in multiple tumor types, including lung [33], brain [35], head and neck [47], liver [45], prostate [51], and cervical cancers [52], chordomas [53], and solid tumors [100]. In addition to cancer imaging, this radiotracer can also be used to image other ischemic conditions, such as cardiac and brain ischemia. ^18^F-FMISO has shown promising results, and efforts continue to refine hypoxia imaging agents, including the development of tracers with faster clearance and greater sensitivity. The potential of these agents not only for static imaging but prognostication may make them an important part of cancer imaging in the future.

### 2.3. Gastrin-Releasing Peptide Receptors (GRPrs) Imaging Agents

Gastrin-releasing peptide receptors (GRPrs) are seven-transmembrane G-protein-coupled receptors that bind to gastrin-releasing peptide (GRP) and belong to the bombesin protein receptor family. Peptide binding to the GRPr initiates a signaling cascade, ultimately resulting in proliferation, mitosis, and differentiation. Importantly, GRPr has been found to be expressed in multiple cancers, including small-cell lung cancer, prostate cancer, breast cancer, gastrinoma, and others [101], making it an attractive target for pan-tumor imaging. Multiple PET imaging agents have been developed for GRPr PET imaging and have progressed into clinical trials.

**Figure 5:**
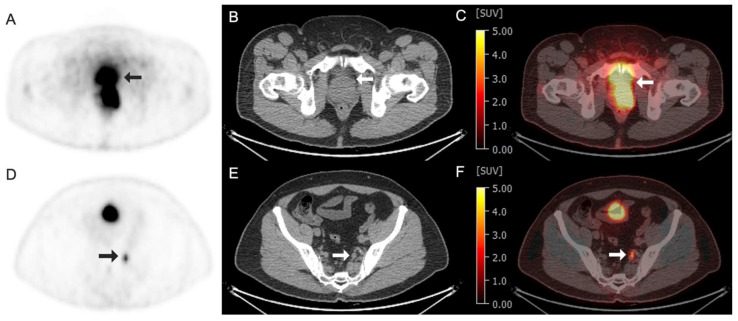
Images obtained from a 60-year-old man with prostate cancer at the level of prostate (**A**–**C**) and internal iliac node (**D**–**F**). (**A**–**C**) Arrows demonstrated an intense radiotracer uptake at the left aspect of the prostate, representing the malignancy. (**A**) Gallium 68 (^68^Ga)-labeled RM2 axial PET image; (**B**) axial CT image; (**C**) axial fused ^68^Ga–RM2 PET/CT image with an SUV max of 24. (**D**–**F**) Arrows demonstrated a radiotracer avid in the sub-centimeter left internal iliac node, subsequently biopsied, and consistent with metastasis. (**D**) ^68^Ga–RM2 axial PET image; (**E**) axial CT image; (**F**) axial fused ^68^Ga–RM2 PET/CT image with an SUV max of 8.4.

^68^Ga-RM2 is a GRPr antagonist that is conjugated to a DOTA molecule to facilitate conjugation chemistry with ^111^In and ^68^Ga for SPECT and PET imaging, respectively, and has shown high tumor to background uptake [102]. Early work established its biodistribution and dosimetry [103], and it has progressed through the phases of clinical trials. In the year of 2024, a study was published in *Lancet Oncology* evaluating its utility compared to MRI in patients with high prostate-specific antigen (PSA) [104]. The study found that 69% of patients had a lesion on ^68^Ga-RM2 PET/MRI, compared to 40% in MRI alone. Authors concluded that this PET agent had better diagnostic performance, and further studies comparing results to PSMA PET are needed to evaluate if either is superior for disease detection [104]. A head-to-head comparison of ^68^Ga-RM2 and ^68^Ga-PSMA-11 PET/CT demonstrated different uptake patterns between the two radiotracers, suggesting expression of the two receptors may be unrelated and that imaging both may provide complementary information to fully characterize the extent of disease [105]. Another clinical trial has evaluated the performance of ^68^Ga-RM2 for detecting prostate cancer at various stages [61], as well as its ability to detect other types of cancers. Ultimately, this body of data will establish when and how this tracer will be used clinically. Figure 5 shows the example ^68^Ga-RM2 clinical images obtained from a 60-year-old man with prostate cancer.

While ^68^Ga-RM2 has emerged as the lead GRPr imaging agent, others have been developed and demonstrated similarly excellent cancer imaging characteristics, including ^64^Cu-Sarcophagine–Bombesin (^64^Cu-SAR-BBN). A study in 2022 of 7 metastatic estrogen receptor positive/progesterone receptor positive/human epidermal growth factor 2 negative (ER+/PR+/HER2-) breast cancer patients using ^64^Cu-SAR-BBN PET/CT evaluated the safety and efficacy of this agent, as well as comparatively evaluated its ability for lesion characterization against ^18^F-FDG. Five of the seven patients had ^64^Cu-SAR-BBN-positive lesions, while ^18^F-FDG detected lesions in all five of these patients. A single patient was ^64^Cu-SAR-BBN-positive/^18^F-FDG-negative. No adverse events were reported in the study. Given the safety of the agent and the findings that suggest there may be biological differences between ^64^Cu-SAR-BBN and ^18^F-FDG positive lesions, the authors concluded that more studies are needed to better understand the utility of this tracer [106]. In parallel, the precursor molecule has been conjugated to a therapeutic radionuclide [107], and positive safety and efficacy in early trials have led to the COMBAT trial, with the first cohort enrolling in early 2024 [108]. Although in the early stages, if results are ultimately positive and have distinct biological activity compared to PSMA-based agents, this therapeutic combination may provide a complementary theranostic combination for patients who do not respond to PSMA radiotherapeutics.

Similarly to the SAR-BBN-based agents, a theranostic pair using the NeoBOMB precursor molecule has been developed and tested through the early stages of clinical trials. Initially tested in an in vivo GRPr-expressing PC-3 prostate cancer model [109], the combination progressed to early clinical trials, which showed tolerable safety and uptake in neoplastic lesions [110]. Currently, ongoing trials are following up on these promising initial results, evaluating the diagnostic performance of ^68^Ga-NeoBOMB1 in malignancies overexpressing GRPr and are performing comparative analyses to assess how results of ^68^Ga-NeoBOMB1 PET/MRI compare to those of ^68^Ga-PSMA-R2 [68].

### 2.4. Integrin Imaging Agents

Integrins are a group of heterodimeric transmembrane glycoproteins, each consisting of 1 of 18 alpha units and 1 of 8 beta units, which are responsible for signal transduction and cellular interactions [111]. The integrin receptor is a practical target for radiopharmaceuticals, given that integrins play key roles in angiogenesis and migration, and the integrin receptor expression is significantly correlated with cancer progression and metastasis [112,113]. Integrins αvβ3 and αvβ6 are overexpressed in a wide variety of tumors and are expressed at substantially lower levels in most normal epithelial cells; this feature makes them attractive radiotheranostics targets of miscellaneous cancers [114]. Binding of αvβ3 to the vitronectin surface is achieved via the RGD (Arg-Gly-Asp) tripeptide, which functions as a core recognition motif. Given the multiple subtypes of integrins, numerous PET imaging agents have been developed. ^18^F-Galacto-RGD was the first PET imaging agent developed to image these molecules about 20 years ago, and, since then, multiple PET imaging agents have been developed and progressed to varying stages of clinical trials. Some of the most promising have emerged for αvβ3 and αvβ6 integrins, which we detail below.

The αvβ3 integrin has emerged as particularly relevant to cancer progression, given its proven role in tumor angiogenesis and invasion [115]. One of the most promising agents to emerge targeting αvβ3 integrin was initially known as ^18^F-AH111585, and subsequently rebranded ^18^F-fluciclatide as it moved into clinical trials. Of note, this agent also images αvβ5 integrin, a second integrin upregulated in tumor angiogenesis. Initial safety and tolerability were established in a cohort of 7 patients with 18 metastatic breast cancer lesions, where the agent was well tolerated and detected all lesions compared to background uptake [116]. A subsequent study of this tracer in renal cell carcinoma and melanoma showed similar excellent tumor-to-background ratios and visualized both primary and metastatic lesions [117]. A phase 1B clinical trial of patients with platinum-resistant/refractory ovarian cancer evaluated the value of ^18^F-fluciclatide to predict response to pazopanib. The study showed that ^18^F-fluciclatide uptake was predictive of long progression-free survival, and that radioligand delivery was reduced following pazopanib administration, indicative of response [118]. Ongoing clinical trials are evaluating how ^18^F-fluciclatide imaging compared directly to ^18^F-FDG in solid tumors and potentially demonstrate non-inferiority of ^18^F-fluciclatide to ^18^F-FDG, laying the groundwork for widespread clinical use of this radiotracer. Wu et al. recently published clinical images using one of these imaging agents, ^18^F-Alphatide II, for the evaluation of imaging axillary lymph nodes in breast cancer patients and compared this to imaging with 18F-FDG [119]. Multiple additional agents targeting αvβ3/αvβ5 utilizing both ^18^F and ^68^Ga, as well as different chemical synthesis strategies, are in various stages of development and may ultimately supplant ^18^F-fluciclatide as the lead agent, but the fundamental importance of integrin-targeting agents comes not from the agent itself, but its ability to give functional and diagnostic information about tumors that can possibly inform clinical decision making.

Other work has focused on the development of αvβ6 imaging agents. Studies have shown that high αvβ6 promotes collagen deposition via the transforming growth factor beta (TGF-β) pathway, and high expression is associated with poor outcomes [120]. One of the most promising agents targeting this heterodimer is ^18^F-αvβ6-BP, a linear peptide based on an earlier molecule A20FMDV2 with chemical modification to decrease off-target radiotracer accumulation [121]. Hausner et al. performed preclinical studies in a mouse model bearing αvβ6-expressing xenografts, followed by first-in-human studies in patients with metastatic lung, colon, breast, or pancreatic cancer. In patients, they observed significant uptake in primary lesions and metastatic sites, including those in the brain, bone, lung, and liver [122]. Continued development of this molecule, including the addition of an albumin moiety and the use of ^64^Cu as the imaging radionuclide have further improved tumor uptake and tracer circulation time, resulting in improved tumor visualization [123].

An alternate αvβ6 imaging agent, ^68^Ga-Trivehexin, was developed and tested in xenograft models and patients with head and neck squamous cell carcinoma, parotid adenocarcinoma, and metastatic pancreatic ductal adenocarcinoma. Data demonstrated that ^68^Ga-Trivehexin had a high affinity for αvβ6 in cell assays and a high tumor-to-background uptake ratio in patients [124]. In a follow-up head-to-head comparison of ^68^Ga-Trivehexin and ^18^F-FDG, ^68^Ga-Trivehexin demonstrated superior sensitivity, specificity, positive predictive value, and negative predictive value compared to ^18^F-FDG. However, authors did not report if these results were statistically significant and concluded that larger-scale studies are needed. Further testing via clinical trials will demonstrate if αvβ6 targeting agents can compete with PET agents against ^18^F-FDG and other pan-tumor markers like those described above.

Apart from the pan-cancer imaging agents discussed above, there are also numerous other emerging pan-cancer targets involved in radiopharmaceutical development. Some of these include C-X-C chemokine receptor type 4 (CXCR4) [125,126,127], urokinase-type plasminogen activator receptor (uPAR) [128,129,130], poly(ADP-ribose) polymerase (PARP) [131,132,133,134], neurotensin receptor 1 (NTSR-1) [135], nectin cell adhesion molecule 4 (Nectin-4) [136], cluster of differentiation 8 (CD8) [137,138,139], programmed cell death 1 ligand 1 (PD-L1) [140,141,142,143,144], and indoleamine 2,3-dioxygenase (IDO) [145] are all being developed and tested. While offering substantial promise to their respective cancer research fields, there are numerous regulatory steps needed before they are available for use.

Thus, it is important to note the status of regulatory approval for radiopharmaceuticals. Zhang et al. have comprehensively reviewed all radiopharmaceutical imaging agents that have been approved worldwide up till the year of 2025. A total of 67 radiopharmaceuticals are currently approved worldwide, of which 54 are used for disease diagnosis and 13 for therapy [146].

Singnurkar et al. have systematically reviewed and compared the diagnostic performance of the traditional FDG PET/CT and FDG PET/MRI in cancer patients in their meta-analysis and demonstrated that FDG PET/MRI showed comparable or superior performance relative to FDG PET/CT across a range of cancers and end points [147]. An in-depth comparative analysis of different imaging modalities, individually or combined, in the context of the novel radiopharmaceuticals would warrant future research endeavors. Recently, Schwenck et al. have proposed a next-generation, multistep approach to collect multimodal imaging data, combined with advanced image analysis based on machine learning and artificial intelligence algorithms, which could enable precision diagnosis and personalized image-guided treatment to be achieved [148].

Finally, validation and clinical qualification of imaging biomarkers are essential steps in translating promising imaging technologies into reliable tools for research and patient care. By demonstrating their technical performance, their association with biological and clinical outcomes, and their suitability for specific uses, imaging biomarkers can significantly improve disease diagnosis, prognosis, treatment selection, and monitoring, ultimately contributing to better patient outcomes.

## 3. Conclusions

Advances in targeted radioligand therapy have already resulted in the development of numerous agents that hold the potential to revolutionize personalized oncology and precision medicine. In addition to providing better lesion characterization, molecular targeted imaging can offer physiologic information about sites of cancer with prognostic value and can direct therapy. Companion radiotherapy agents deliver a radioactive payload to all sites of disease and have limited side effects. Agents targeting FAP, hypoxia, GRPr, and integrins are among those that have progressed furthest in clinical trials due to their excellent avidity for tumors and pan-cancer targeting properties, and are poised to enter clinical use soon. Together, new PET imaging agents and their companion radiotherapies will play an increasingly significant role in patient stratification and treatment, resulting in improved therapeutic outcomes.

Despite the promising future of targeted radioligand therapy, challenges remain. Herrmann et al. have thoroughly discussed and categorized them as follows: 1) technical or organizational (absence of interdisciplinary teams, small workforce, bottlenecks in radioisotope availability, uneven global availability, regulatory challenges); 2) economic (high development cost, ill-defined reimbursement, insufficient research funding, competing technologies, global differences); and 3) biomedical (few available drugs, absence of large-scale prospective trials, largely unexplored combination treatments) [149]. In order to continue the progress of this evolving therapeutic approach, challenges need to be carefully addressed in multidisciplinary, multi-center, and cross-regional teams and must subsequently be widely implemented.

## Figures and Tables

**Table 1:** Ongoing clinical trials evaluating fibroblast activation protein targeting agents.

Study Title	Primary Purpose	Study Type, Phase	ClinicalTrials.gov ID	Status	No. of Patients
^18^F-FDG Versus ^68^Ga-FAPI-46 as PET Tracer in ER-positive **Breast Cancer**	Diagnostic	Interventional, Phase 2	NCT06335069 [9]	Not Yet Recruiting	10
^68^Ga-FAPI PET in Evaluation of the Patient With Known or Suspected **Lung Cancer**: Comparison With Standard ^18^F-FDG PET	Diagnostic	Interventional, N/A	NCT05617742 [10]	Recruiting	103
A Phase 2, Multicenter, Single Arm, Open Label Non-Randomized Study of ^68^Ga-FAPI-46 PET in Patients With Resectable or Borderline Resectable **Pancreatic Ductal Carcinoma**	Diagnostic	Interventional, Phase 2	NCT05262855 [11]	Active, Not Recruiting	60
Initial Staging of Lobular **Breast Carcinoma**: Head to Head Comparison of ^68^Ga-FAPI-46 and ^18^F-FDG PET/CT	Diagnostic	Interventional, N/A	NCT05931302 [12]	Recruiting	40
^68^Ga-FAPI-46 PET in **Pancreaticobiliary Cancers**: A Pharmacokinetics, Repeatability and Diagnostic Accuracy Study	Diagnostic	Interventional, N/A	NCT05957250 [13]	Recruiting	63
^68^Ga-FAPI-46 PET for Imaging of **FAP Expressing Cancer**: A Single-center Prospective Interventional Single-arm Clinical Trial	Diagnostic	Interventional, Phase 2	NCT05160051 [14]	Completed on 6 March 2024	158
^68^Ga-FAPI-46 PET/CT: The Diagnostic Accuracy for Primary Staging and Re-staging of Patients With **Ovarian Cancer**	Diagnostic	Interventional, Phase 2	NCT05903807 [15]	Recruiting	50
Single Centre Prospective Evaluation of ^68^Ga-FAPI PET/MRI in **Hepatocellular Carcinoma**	Diagnostic	Interventional, Phase 1	NCT05687747 [16]	Recruiting	100
(FAPI-CUP) Evaluating FAPI as a Novel Radiopharmaceutical Targeting Cancer-associated Fibroblasts for the Diagnosis of Patients With **Cancer of Unknown Primary**	Diagnostic	Interventional, N/A	NCT05263700 [17]	Unknown	150
PET Imaging of **Tumors in Pancreas, Bile Ducts, Stomach and Ovaries** by a Novel Tracer, ^68^Ga-FAPI-46	Diagnostic	Interventional, Phase 1	NCT05172310 [18]	Recruiting	410
PET Biodistribution Study of ^68^Ga-FAPI-46 in Patients With **Sarcoma**: An Exploratory Biodistribution Study With Histopathology Validation	Diagnostic	Interventional, Phase 1	NCT04457258 [19]	Recruiting	30
PET Biodistribution Study of ^68^Ga-FAPI-46 in Patients With **Prostate Cancer**: A Prospective Exploratory Biodistribution Study With Histopathology Validation	Diagnostic	Interventional, Phase 1	NCT04457232 [20]	Active, Not Recruiting	30
(MI-CISDIR) Multimodal Imaging With FAPI-PET/MRI in **Breast Carcinoma-In Situ** for Detection of Occult Invasive Cancer	Diagnostic	Interventional, Phase 2	NCT06540872 [21]	Not Yet Recruiting	30
^68^Ga-FAPI-46 PET/CT for Predicting Histological Response to Neoadjuvant Chemo-immunotherapy in Triple-negative **Breast Cancer**	Diagnostic	Interventional, N/A	NCT06349512 [22]	Recruiting	60
Evaluation of ^68^Ga-FAPI-46 and ^18^F-FDG PET/CT Imaging for Detecting Recurrent Tumor Lesions in Patients of **Ovarian Cancer** With CA125 Elevation From Complete Response After Therapy	Diagnostic	Interventional, N/A	NCT06232122 [23]	Recruiting	45
(PARADISE) ^68^Ga-FAPI PET/CT Imaging in **Chronic Inflammatory and Fibrotic Diseases**	Diagnostic	Interventional, N/A	NCT06275477 [24]	Not Yet Recruiting	390
(FAPI ILD) PET Study of ^68^Ga-FAPi-46 in Patients With **Interstitial Lung Disease**: An Exploratory Biodistribution Study With Histopathology Validation	Diagnostic	Interventional, Phase 1	NCT05365802 [25]	Recruiting	30
(HEFITEP) Pilot Study of **Liver Fibrosis** Stage Assessment by Fibroblast Activation Protein Imaging (^68^Ga-FAPI-46 TEP/TDM) in Patients With Biopsy for Suspected or Proven Nonalcoholic Steatohepatitis	Diagnostic	Interventional, Phase 2	NCT06160271 [26]	Not Yet Recruiting	72
(PIMAFI) FAPi-PET Imaging of in Vivo Fibrosis in **Inflammatory Bowel Disease** Patients	Diagnostic	Interventional, Phase 2	NCT06604260 [27]	Recruiting	20
(LuMIERE) A Phase 1/2, Multicenter, Open-label, Non-randomized Study to Investigate Safety and Tolerability, Pharmacokinetics, Dosimetry, and Preliminary Activity of ^177^Lu-FAP-2286 in Patients With an **Advanced Solid Tumor**	Therapeutic (Treatment)	Interventional, Phase 1 and 2	NCT04939610 [28]	Recruiting	222
Imaging of **Solid Tumors** Using FAP-2286	Diagnostic	Interventional, Phase 1	NCT04621435 [29]	Recruiting	131
Evaluating the Potential Usefulness of ^68^Ga-FAP-2286 PET/CT in Patients With **Various Types of Cancer** and Compared With ^18^F-FDG PET/CT	Diagnostic	Interventional, N/A	NCT05392205 [30]	Completed on 31 October 2022	67
^68^Ga-FAPI-RGD PET/CT for Dual Integrin αvβ3 and FAP-targeted Imaging in Patients With **Various Types of Cancer** and Compared With ^18^F-FDG	Diagnostic	Interventional, N/A	NCT05543317 [31]	Completed on 31 December 2022	27
^68^Ga-FAPI-LM3 PET/CT Imaging in Patients With **FAP/SSTR2 Positive Disease** and Compared With ^18^F-FDG	Diagnostic	Interventional, N/A	NCT05873777 [32]	Recruiting	30

**Table 2:** Ongoing clinical trials evaluating hypoxia imaging agents.

Study Title	Primary Purpose	Study Type, Phase	ClinicalTrials.gov ID	Status	No. of Patients
Randomized Phase II Trial of Individualized Adaptive Radiotherapy Using During-Treatment FDG-PET/CT and Modern Technology in Locally Advanced Non-Small Cell **Lung Cancer**	Therapeutic (Treatment)	Interventional, Phase 2	NCT01507428 [33]	Unknown	138
(MISOGLIO) Methodological Evaluation of ^18^F-FMISO PET-CT for Non-Operated **Glioblastoma**	Health Services Research	Interventional, Phase 2	NCT00906893 [34]	Completed in January 2013	14
Multicenter, Phase II Assessment of Tumor Hypoxia in **Glioblastoma** Using ^18^F-FMISO With PET and MRI	Diagnostic	Interventional, Phase 2	NCT00902577 [35]	Completed on 31 February 2018	50
(FMISOPETSCS) PET with ^18^F-FMISO in High Grade **Gliomas**: Assessment of Tumor Hypoxia and Effect of Spinal Cord Stimulation	Diagnostic	Interventional, Phase 2	NCT01868906 [36]	Terminated	6
Pilot Study of ^18^F-FMISO PET/CT and MRI Imaging to Explore Tissue Hypoxia and Arteriovenous Shunting in Subjects With Recurrent **Glioblastoma** Before and After Bevacizumab Treatment	Diagnostic	Interventional, Phase 1	NCT03573986 [37]	Terminated	2
Feasibility of ^18^F-FMISO in Assessment of **Malignant Brain Tumors**	Diagnostic	Interventional, Phase 2	NCT03649880 [38]	Recruiting	50
Assessment of Primary and Metastatic **Brain Tumor** Hypoxia With ^18^F-FMISO, ^18^F-FDG and H_2_^15^O	Diagnostic	Interventional, Phase 1	NCT01246869 [39]	Terminated	2
A Phase-I Trial for Simultaneous Imaging of Tumor Hypoxia and Proliferation in Patients With Treatment—Naïve High-Grade **Glioma**	Diagnostic	Interventional, Phase 1	NCT04309552 [40]	Suspended	30
^18^F-FMISO PET Guided Dose Escalation in **Nasopharyngeal Carcinoma**—a Feasibility and Planning Study	Diagnostic	Interventional, N/A	NCT04995185 [41]	Completed on 30 December 2020	9
(MISORL) Prognostic Evaluation of ^18^F-FMISO PET-CT in **Head and Neck Squamous Cell Carcinomas**	Health Services Research	Interventional, N/A	NCT01235052 [42]	Completed in July 2014	16
Clinical Feasibility Study of Hypoxia Imaging-Guided Intensity Modulated Radiotherapy on the Individualized Radiotherapy of **Nasopharyngeal Carcinoma**	Observational	Observational (Case–Control)	NCT02089204 [43]	Unknown	300
(GKH-TMM) Moderate Whole Body Hyperthermia for Patients Undergoing Re-irradiation for **Head and Neck Cancer** -Influence on the Tumor Microenvironment	Therapeutic (Treatment)	Interventional, Phase 1	NCT03547388 [44]	Completed on 4 May 2020	10
Assessment of **Hypoxia Before Radioembolization** Treatment With ^18^F-FMISO PET	Observational	Observational (Cohort)	NCT06027021 [45]	Completed on 15 July 2024	64
Hypoxic Changes in **Hepatocellular Carcinoma** Following Trans-arterial Chemo Embolization and Stereotactic Radiation: ^18^F-FMISO Imaging	Diagnostic	Interventional, Phase 2	NCT03303469 [46]	Terminated	3
A Pilot Study of Radiation De-Escalation for P16 Negative **Oropharyngeal Cancer** and P16-Negative or Positive **Laryngeal and Hypopharyngeal Cancers**	Therapeutic (Treatment)	Interventional, Phase 2	NCT05544136 [47]	Recruiting	12
Assessment of Treatment-Induced Tissue Hypoxia After Transcatheter Arterial Embolization of **Hepatocellular Carcinoma**: A Feasibility Study With ^18^F-FMISO PET/CT	Diagnostic	Interventional, Phase 2	NCT02695628 [48]	Completed on 31 October 2018	5
Molecular Imaging of the Hypoxic Tumor Microenvironment to Predict Response to Yttirum-90 Selective Internal Radiation Therapy in **Hepatocellular Carcinoma**-Pilot Study	Diagnostic	Interventional, Phase 1	NCT05250895 [49]	Completed on 26 February 2025	20
(HYPOXProstat) Evaluation of Hypoxia by PET with ^18^F-FMISO During Radiation Therapy of **Prostate Cancer**	Diagnostic	Interventional, Phase 2	NCT01898065 [50]	Completed in February 2015	20
(LuMISO) Effect of ^18^F-FMISO PET Imaging on Evaluation of Hypoxia Before ^177^Lu-PSMA Treatment for **Prostate Cancer**	Diagnostic	Interventional, N/A	NCT06433063 [51]	Recruiting	30
A Phase 2 Study of PET Imaging With ^18^F-FMISO and ^18^F-FDG for Assessment of Tumor Hypoxia in **Cervical Cancer**	Diagnostic	Interventional, Phase 2	NCT00559377 [52]	Completed in May 2015	16
Hypoxia-PET and Intensity Modulated Proton Therapy Dose Painting in Patients With **Chordomas**: A Pilot Study	Diagnostic	Interventional, N/A	NCT00713037 [53]	Completed in June 2016	20
A Phase 2 Study of PET Imaging with ^18^F-FMISO and ^18^F-FDG for Assessment of Tumor Hypoxia in **Soft Tissue Sarcoma**	Diagnostic	Interventional, Phase 2	NCT01169350 [54]	Terminated	8
(FIPOXY) Evaluation of the Value of ^18^F-FMISO PET Hypoxia Imaging in **Idiopathic Pulmonary Fibrosis**—A Non-randomized Proof-of-concept Study Comparing Patients with Idiopathic Pulmonary Fibrosis and Healthy Subjects	Diagnostic	Interventional, Phase 1	NCT05331729 [55]	Completed on 20 February 2025	20

**Table 3:** Ongoing clinical trials evaluating gastrin-releasing peptide receptor imaging agents.

Study Title	Primary Purpose	Study Type, Phase	ClinicalTrials.gov ID	Status	No. of Patients
^68^Ga-RM2 PET/MRI in the Evaluation of Patients With Biochemical Recurrence of **Prostate Cancer** and Non-contributory CT Scans	Diagnostic	Interventional, Phase 2 and 3	NCT02624518 [56]	Completed in November 2022	122
A Phase II Study of ^68^Ga-RM2 for PET/CT of GRPr Expression in **Prostate Cancer**	Diagnostic	Interventional, Phase 2	NCT02559115 [57]	Completed on 18 June 2020	19
^68^Ga-RM2 PET/CT for Detection of Regional Nodal and Distant Metastases in Patients with Intermediate and High-Risk **Prostate Cancer**	Diagnostic	Interventional, Phase 2	NCT03113617 [58]	Completed on 19 December 2021	44
Evaluation of the LightPath^®^ Imaging System and the PET Tracer ^68^Ga-RM2 in Wide Local Excision for **Breast Cancer**	Diagnostic	Interventional, Phase 3	NCT03731026 [59]	Unknown	80
(PROSTATEP) Exploratory, Single-institution Study, Comparing ^68^Ga-RM2 PET/CT Versus ^68^Ga-PSMA-617 PET/CT in Patients Diagnosed with Intermediate Risk **Prostate Cancer** Candidates for Radical Prostatectomy	Therapeutic (Treatment)	Interventional, Phase 2	NCT03606837 [60]	Completed on 11 July 2023	15
Phase II Prospective Monocentric Study on **Prostate Cancer** Restaging by Using PET/MR with Innovative Radiotracers	Diagnostic	Interventional, Phase 2	NCT05806853 [61]	Completed on 5 October 2022	60
A Pilot Study of ^68^Ga-RM2 PET/MRI in the Evaluation of Patients with Estrogen Receptor-Positive **Breast Cancer**	Diagnostic	Interventional, Phase 1 and 2	NCT03831711 [62]	Completed on 15 November 2021	5
(SABRE) ^64^Cu-SAR-BBN PET: A Phase 2 Study of Participants with PSMA-negative Biochemical Recurrence of **Prostate Cancer**	Diagnostic	Interventional, Phase 2	NCT05407311 [63]	Completed on 13 May 2024	53
(BOP) Assessment of the Diagnostic Value of ^64^Cu-SAR-BBN PET Imaging for Men With Negative PSMA PET in **Prostate Cancer**	Diagnostic	Interventional, Phase 2	NCT05613842 [64]	Completed on 6 June 2023	30
(COMBAT) A Phase I/IIa Theranostic Study of ^64^Cu-SAR-BBN and ^67^Cu-SAR-BBN for Identification and Treatment of GRPR-expressing Metastatic Castrate Resistant **Prostate Cancer** in Patients Who Are Ineligible for Therapy With ^177^Lu-PSMA-617	Therapeutic (Treatment)	Interventional, Phase 1 and 2	NCT05633160 [65]	Recruiting	38
(MITIGATE-NeoBOMB1) A Phase I/IIa Study to Evaluate Safety, Biodistribution, Dosimetry, and Preliminary Diagnostic Performance of ^68^Ga-NeoBOMB1 in Patients with Advanced TKI-treated **Gastrointestinal Stromal Tumor** Using PET/CT	Diagnostic	Interventional, Phase 1 and 2	NCT02931929 [66]	Completed on 9 April 2019	9
(NeoFIND) Phase II Study of Preliminary Diagnostic Performance of ^68^Ga-NeoBOMB1 in Adult Patients with **Malignancies Known to Overexpress Gastrin Releasing Peptide Receptor**	Diagnostic	Interventional, Phase 2	NCT03724253 [67]	Terminated	19
^68^Ga-NeoBOMB1 and ^68^Ga-PSMA R2 PET/MRI in the Evaluation of Patients with Biochemical Recurrence of **Prostate Cancer**	Diagnostic	Interventional, Phase 2	NCT03698370 [68]	Completed on 11 February 2022	27
(NeoRay) A Phase I/IIa Open-label, Multi-center Study to Evaluate the Safety, Tolerability, Whole-body Distribution, Radiation Dosimetry and Anti-tumor Activity of ^177^Lu-NeoB Administered in Patients with **Advanced Solid Tumors** Known to Overexpress Gastrin-releasing Peptide Receptor	Therapeutic (Treatment)	Interventional, Phase 1 and 2	NCT03872778 [69]	Active, Not Recruiting	51
A Phase I/II, Open-label, Multi-center Trial of ^177^Lu-NeoB in Combination With Capecitabine in Adult Patients With Gastrin Releasing Peptide Receptor Positive, Estrogen Receptor-positive, Human Epidermal Growth Factor Receptor-2 Negative Metastatic **Breast Cancer** After Progression on Previous Endocrine Therapy in Combination With a CDK4/6 Inhibitor	Therapeutic (Treatment)	Interventional, Phase 1 and 2	NCT06247995 [70]	Recruiting	58
A Phase Ib Dose Finding Study Assessing Safety and Activity of ^177^Lu-NeoB in Combination With Ribociclib and Fulvestrant in Participants With Estrogen Receptor Positive, Human Epidermal Growth Factor Receptor-2 Negative and Gastrin Releasing Peptide Receptor Positive Advanced **Breast Cancer** Experiencing Early Relapse From (Neo)Adjuvant Endocrine Therapy or Who Have Progressed on Endocrine Therapy in Combination With a CDK4/6 Inhibitor for Advanced Disease	Therapeutic (Treatment)	Interventional, Phase 1	NCT05870579 [71]	Recruiting	48
Phase Ib Dose Finding Study Assessing Safety and Activity of ^177^Lu-NeoB in Combination With Radiotherapy and Temozolomide in Subjects With Newly Diagnosed **Glioblastoma** and as a Single Agent in Recurrent Glioblastoma	Therapeutic (Treatment)	Interventional, Phase 1	NCT05739942 [72]	Recruiting	48
Assessment of the Diagnostic and Theranostic Potential of ^68^Ga-Bombesin PET/CT (NeoB) Imaging for Staging of ER/PR + HER2- **Breast Cancer** Patients with Metastatic Disease: Comparison to Conventional Imaging	Diagnostic	Interventional, Phase 2	NCT05889728 [73]	Unknown	20
(NEPC) A Phase I, Open-label, Multi-center Exploratory Safety and Efficacy Study With PSMA, SSTR2 and GRPR Targeted Radioligand Therapy in Metastatic **Neuroendocrine Prostate Cancer**	Therapeutic (Treatment)	Interventional, Phase 1	NCT06379217 [74]	Recruiting	36

**Table 4:** Ongoing clinical trials evaluating integrin imaging agents.

Study Title	Primary Purpose	Study Type, Phase	ClinicalTrials.gov ID	Status	No. of Patients
A Phase 2, Open-label, Proof-of-concept Study to Assess the Ability to Detect **Tumors and Angiogenesis** Via the Expression of ανβ3/5 Integrin Receptors by ^18^F-AH-111585 PET Imaging	Diagnostic	Interventional, Phase 2	NCT00565721 [75]	Completed in September 2012	33
A Test–Retest Study to Assess Reproducibility of ^18^F Uptake by **Solid Tumors** Using PET Imaging Following Intravenous Administration of ^18^F-AH111585 Injection	Diagnostic	Interventional, Phase 2	NCT00918281 [76]	Completed in October 2011	70
(PAZPET-1) Phase 1b Exploratory Study of ^18^F-Fluciclatide-PET as a Marker of Angiogenic Response to Combination Therapy With the Pan-VEGF Inhibitor, Pazopanib, and Weekly Paclitaxel in Platinum Resistant **Ovarian Cancer**	Therapeutic (Treatment)	Interventional, Phase 1	NCT01608009 [77]	Completed in April 2016	16
(GRGDG) Diagnostic Performance and Evaluation Efficacy of Brain ^68^Ga-BNOTA-PRGD2 PET/CT in Pre-surgery **Glioma** Patients	Diagnostic	Interventional, Phase 1	NCT01801371 [78]	Unknown	30
(GRGDLC) Radiation Dosimetry, Plasma Pharmacokinetics, Biodistribution, Safety and Diagnostic Performance of ^68^Ga-BNOTA-PRGD2 in Healthy Volunteers and **Lung Cancer** Patients	Diagnostic	Interventional, Phase 1	NCT01527058 [79]	Unknown	100
Diagnosis of **Metastatic Tumors** on ^68^Ga-FAPI-RGD PET-CT and Radioligand Therapy	Therapeutic (Treatment)	Interventional, Phase 1	NCT06638034 [80]	Recruiting	15
^68^Ga-FAPI-RGD PET/CT for Dual Integrin αvβ3 and FAP-targeted Imaging in Patients With **Various Types of Cancer** and Compared With ^18^F-FDG	Diagnostic	Interventional, N/A	NCT05543317 [31]	Completed on 31 December 2022	27
A Novel Dual-Targeting Molecular Probe TATE-RGD for the Diagnostic Integration of **SSTR2 and αvβ3 Positive Tumors**	Diagnostic	Interventional, Phase 1	NCT06632860 [81]	Recruiting	40
^68^Ga-RM26-RGD PET/CT Imaging in the **GRPR and αvβ3 Positive Tumor** Patients	Diagnostic	Interventional, Phase 1	NCT05549024 [82]	Recruiting	90
Therapeutic Efficiency and Response to ^177^Lu-AB-3PRGD2 in Patients with Integrin **αVβ3 Positive Tumors**	Therapeutic (Treatment)	Interventional, Phase 1	NCT05013086 [83]	Recruiting	10
Phase 1/2 ^18^F-FPPRGD2 PET/CT or PET/MRI Imaging of αvβ3 Integrins Expression as a Biomarker of **Angiogenesis**	Diagnostic	Interventional, Phase 1 and 2	NCT01806675 [84]	Completed in April 2019	25
**Biodistribution and Safety** of the PET Probes ^18^F-FPRGD2 and ^18^F-FPPRGD2	Diagnostic	Interventional, Phase 1	NCT01383135 [85]	Completed in December 2013	27

## Supplementary Materials

The following supporting information can be downloaded at: https://www.mdpi.com/article/10.3390/tomography11080083/s1. Figure S1: The flow diagram illustrating the process of literature identification, the databases searched, and the inclusion and exclusion criteria applied during the screening and eligibility assessment of milestone publications and trials for this narrative review.

## Abbreviations

RLT	Radioligand Therapy
PSA	Prostate-specific Antigen
PSMA	Prostate-specific Membrane Antigen
PET/CT	Positron Emission Tomography/Computed Tomography
MIP	Maximum Intensity Projection
SUV	Standard Uptake Value
CAF	Cancer-associated Fibroblast
FAP(I)	Fibroblast Activation Protein (Inhibitor)
GRPr	Gastrin-releasing Peptide Receptor
FDG	Fluorodeoxyglucose
FMISO	Fluoromisonidazole
EDTMP	Ethylene Diamine Tetramethylene Phosphonate
SSTR	Somatostatin Receptor
CXCR4	C-X-C Chemokine Receptor Type 4
uPAR	Urokinase-type Plasminogen Activator Receptor
PARP	Poly(ADP-Ribose) Polymerase
NTSR-1	Neurotensin Receptor 1
Nectin-4	Nectin Cell Adhesion Molecule 4
CD8	Cluster of Differentiation 8
IDO	Indoleamine 2:3-Dioxygenase
ER	Estrogen Receptor
PR	Progesterone Receptor
HER2	Human Epidermal Growth Factor 2
PD-L1	Programmed Cell Death 1 Ligand 1
CTLA-4	Cytotoxic T-Lymphocyte-associated Protein 4
SAR-BBN	Sarcophagine–Bombesin
RGD	Arginine–Glycine–Aspartate
TGF-β	Transforming Growth Factor Beta

## References

[1] Hertz S., Roberts A. (1946). Radioactive iodine in the study of thyroid physiology; the use of radioactive iodine therapy in hyperthyroidism. J. Am. Med. Assoc..

[2] Shimoni A., Zwas S.T., Oksman Y., Hardan I., Shem-Tov N., Yerushalmi R., Avigdor A., Ben-Bassat I., Nagler A. (2007). Yttrium-90-ibritumomab tiuxetan (Zevalin) combined with high-dose BEAM chemotherapy and autologous stem cell transplantation for chemo-refractory aggressive non-Hodgkin’s lymphoma. Exp. Hematol..

[3] Loh K.C., Fitzgerald P.A., Matthay K.K., Yeo P.P., Price D.C. (1997). The treatment of malignant pheochromocytoma with iodine-131 metaiodobenzylguanidine (131I-MIBG): A comprehensive review of 116 reported patients. J. Endocrinol. Investig..

[4] Anderson P.M., Wiseman G.A., Dispenzieri A., Arndt C.A., Hartmann L.C., Smithson W.A., Mullan B.P., Bruland O.S. (2002). High-dose samarium-153 ethylene diamine tetramethylene phosphonate: Low toxicity of skeletal irradiation in patients with osteosarcoma and bone metastases. J. Clin. Oncol..

[5] Longo J., Lutz S., Johnstone C. (2013). Samarium-153-ethylene diamine tetramethylene phosphonate, a beta-emitting bone-targeted radiopharmaceutical, useful for patients with osteoblastic bone metastases. Cancer Manag. Res..

[6] Robinson R.G., Preston D.F., Schiefelbein M., Baxter K.G. (1995). Strontium 89 therapy for the palliation of pain due to osseous metastases. JAMA.

[7] Strosberg J., El-Haddad G., Wolin E., Hendifar A., Yao J., Chasen B., Mittra E., Kunz P.L., Kulke M.H., Jacene H. (2017). Phase 3 Trial of (177)Lu-Dotatate for Midgut Neuroendocrine Tumors. N. Engl. J. Med..

[8] Jones W., Griffiths K., Barata P.C., Paller C.J. (2020). PSMA Theranostics: Review of the Current Status of PSMA-Targeted Imaging and Radioligand Therapy. Cancers.

[9] 18F-FDG Versus 68Ga-FAPI-46 as PET Tracer in ER-positive Breast Cancer—A Pilot Study. https://clinicaltrials.gov/study/NCT06335069.

[10] 68Ga-FAPI PET in Evaluation of the Patient with Known or Suspected Lung Cancer: Comparison with Standard 18F-FDG PET. https://clinicaltrials.gov/study/NCT05617742.

[11] A Phase 2, Multicenter, Single-Arm, Open-Label Non-Randomized Study of 68Ga-FAPI-46 PET in Patients with Resectable or Borderline Resectable Pancreatic Ductal Carcinoma. https://clinicaltrials.gov/study/NCT05262855.

[12] Initial Staging of Lobular Breast Carcinoma: Head-to-Head Comparison of 68Ga-FAPI-46 and 18F-FDG PET/CT. https://clinicaltrials.gov/study/NCT05931302.

[13] 68Ga-FAPI-46 Positron Emission Tomography in Pancreaticobiliary Cancers: A Pharmacokinetics, Repeatability and Diagnostic Accuracy Study. https://clinicaltrials.gov/study/NCT05957250.

[14] 68Ga-FAPI-46 PET for Imaging of FAP Expressing Cancer: A Single-center Prospective Interventional Single-arm Clinical Trial. https://clinicaltrials.gov/study/NCT05160051.

[15] 68Ga-FAPI-46 PET/CT: The Diagnostic Accuracy for Primary Staging and Re-staging of Patients with Ovarian Cancer. https://clinicaltrials.gov/study/NCT05903807.

[16] Single Centre Prospective Evaluation of 68Ga-FAPI PET/MRI in Hepatocellular Carcinoma. https://clinicaltrials.gov/study/NCT05687747.

[17] FAPI-CUP-Evaluating FAPI as a Novel Radiopharmaceutical Targeting Cancer-associated Fibroblasts for the Diagnosis of Patients with Cancer of Unknown Primary. https://clinicaltrials.gov/study/NCT05263700.

[18] PET Imaging of Tumors in Pancreas, Bile Ducts, Stomach and Ovaries by a Novel Tracer, 68Ga-FAPI-46. https://clinicaltrials.gov/study/NCT05172310.

[19] PET Biodistribution Study of 68Ga-FAPI-46 in Patients with Sarcoma: An Exploratory Biodistribution Study with Histopathology Validation. https://clinicaltrials.gov/study/NCT04457258.

[20] PET Biodistribution Study of 68Ga-FAPI-46 in Patients with Prostate Cancer: A Prospective Exploratory Biodistribution Study with Histopathology Validation. https://clinicaltrials.gov/study/NCT04457232.

[21] (MI-CISDIR) Multimodal Imaging with FAPI-PET/MRI in Breast Carcinoma-In-Situ for Detection of Occult Invasive Cancer. https://clinicaltrials.gov/study/NCT06540872.

[22] 68Ga-FAPI-46 PET/CT for Predicting Histological Response to Neoadjuvant Chemo-immunotherapy in Triple-negative Breast Cancer. https://clinicaltrials.gov/study/NCT06349512.

[23] Evaluation of 68Ga-FAPI-46 and 18F-FDG PET/CT Imaging for Detecting Recurrent Tumor Lesions in Patients of Ovarian Cancer with CA125 Elevation From Complete Response After Therapy. https://clinicaltrials.gov/study/NCT06232122.

[24] (PARADISE) 68Ga-FAPI PET/CT Imaging in Chronic Inflammatory and Fibrotic Diseases. https://clinicaltrials.gov/study/NCT06275477.2024.

[25] (FAPI ILD) PET Study of 68Ga-FAPi-46 in Patients with Interstitial Lung Disease: An Exploratory Biodistribution Study with Histopathology Validation. https://clinicaltrials.gov/study/NCT05365802.

[26] (HEFITEP) Pilot Study of Liver Fibrosis Stage Assessment by Fibroblast Activation Protein Imaging (68Ga-FAPI-46 TEP/TDM) in Patients with Biopsy for Suspected or Proven Nonalcoholic Steatohepatitis. https://clinicaltrials.gov/study/NCT06160271.

[27] (PIMAFI) FAPi-PET Imaging of in Vivo Fibrosis in Inflammatory Bowel Disease Patients. https://clinicaltrials.gov/study/NCT06604260.

[28] (LuMIERE) A Phase 1/2, Multicenter, Open-label, Non-randomized Study to Investigate Safety and Tolerability, Pharmacokinetics, Dosimetry, and Preliminary Activity of 177Lu-FAP-2286 in Patients with an Advanced Solid Tumor. https://clinicaltrials.gov/study/NCT04939610.

[29] Imaging of Solid Tumors Using FAP-2286. https://clinicaltrials.gov/study/NCT04621435.

[30] Evaluating the Potential Usefulness of 68Ga-FAP-2286 PET/CT in Patients with Various Types of Cancer and Compared with 18F-FDG PET/CT. https://clinicaltrials.gov/study/NCT05392205.

[31] 68Ga-FAPI-RGD PET/CT for Dual Integrin αvβ3 and FAP-targeted Imaging in Patients with Various Types of Cancer and Compared with 18F-FDG. https://clinicaltrials.gov/study/NCT05543317.

[32] 68Ga-FAPI-LM3 PET/CT Imaging in Patients with FAP/SSTR2 Positive Disease and Compared with 18F-FDG. https://clinicaltrials.gov/study/NCT05873777.

[33] Randomized Phase II Trial of Individualized Adaptive Radiotherapy Using During-Treatment FDG-PET/CT and Modern Technology in Locally Advanced Non-Small Cell Lung Cancer (NSCLC). https://clinicaltrials.gov/study/NCT01507428.

[34] (MISOGLIO) Methodological Evaluation of 18F-FMISO PET-CT for Non-Operated Glioblastoma. https://clinicaltrials.gov/study/NCT00906893.

[35] Multicenter, Phase II Assessment of Tumor Hypoxia in Glioblastoma Using 18F-Fluoromisonidazole (FMISO) with PET and MRI. https://clinicaltrials.gov/study/NCT00902577.

[36] (FMISOPETSCS) PET with 18F-FMISO in High Grade Gliomas: Assessment of Tumor Hypoxia and Effect of Spinal Cord Stimulation. https://clinicaltrials.gov/study/NCT01868906.

[37] Pilot Study of 18F-FMISO PET/CT and MRI Imaging to Explore Tissue Hypoxia and Arteriovenous Shunting in Subjects with Recurrent Glioblastoma Before and After Bevacizumab Treatment. https://clinicaltrials.gov/study/NCT03573986.

[38] Feasibility of 18F-FMISO in Assessment of Malignant Brain Tumors. https://clinicaltrials.gov/study/NCT03649880.

[39] Assessment of Primary and Metastatic Brain Tumor Hypoxia with 18F-FMISO, 18F-FDG and H215O. https://clinicaltrials.gov/study/NCT01246869.

[40] A Phase-I Trial for Simultaneous Imaging of Tumor Hypoxia and Proliferation in Patients with Treatment-Naïve High-Grade Glioma. https://clinicaltrials.gov/study/NCT04309552.

[41] 18F-FMISO PET Guided Dose Escalation in Nasopharyngeal Carcinoma—A Feasibility and Planning Study. https://clinicaltrials.gov/study/NCT04995185.

[42] (MISORL) Prognostic Evaluation of 18F-FMISO PET-CT in Head and Neck Squamous Cell Carcinomas. https://clinicaltrials.gov/study/NCT01235052.

[43] Clinical Feasibility Study of Hypoxia Imaging -Guided intensity modulated radiotherapy on the Individualized Radiotherapy of Nasopharyngeal Carcinoma. https://clinicaltrials.gov/study/NCT02089204.

[44] (GKH-TMM) Moderate Whole Body Hyperthermia for Patients Undergoing Re-irradiation for Head and Neck Cancer -Influence on the Tumor Microenvironment. https://clinicaltrials.gov/study/NCT03547388.

[45] Assessment of Hypoxia Before Radioembolization Treatment with 18F-FMISO PET. https://clinicaltrials.gov/study/NCT06027021.

[46] Hypoxic Changes in Hepatocellular Carcinoma Following Trans-arterial Chemo Embolization and Stereotactic Radiation: 18F-FMISO Imaging. https://clinicaltrials.gov/study/NCT03303469.

[47] A Pilot Study of Radiation De-Escalation for P16 Negative Oropharyngeal Cancer and P16-Negative or Positive Laryngeal and Hypopharyngeal Cancers. https://clinicaltrials.gov/study/NCT05544136.

[48] Assessment of Treatment-Induced Tissue Hypoxia After Transcatheter Arterial Embolization of Hepatocellular Carcinoma: A Feasibility Study with 18F-FMISO PET/CT. https://clinicaltrials.gov/study/NCT02695628.

[49] Molecular Imaging of the Hypoxic Tumor Microenvironment to Predict Response to Yttirum-90 Selective Internal Radiation Therapy in Hepatocellular Carcinoma-Pilot Study. https://clinicaltrials.gov/study/NCT05250895.

[50] (HYPOXProstat) Evaluation of Hypoxia by PET with 18F-FMISO During Radiation Therapy of Prostate Cancer. https://clinicaltrials.gov/study/NCT01898065.

[51] (LuMISO) Effect of 18F-FMISO PET Imaging on Evaluation of Hypoxia Before Lu-177 PSMA Treatment for Prostate Cancer. https://clinicaltrials.gov/study/NCT06433063.

[52] A Phase 2 Study of Positron Emission Tomography Imaging with [18F]-Fluoromisonidazole (FMISO) and [18F]-Fluorodeoxyglucose (FDG) for Assessment of Tumor Hypoxia in Cervical Cancer. https://clinicaltrials.gov/study/NCT00559377.

[53] Hypoxia-PET and Intensity Modulated Proton Therapy Dose Painting in Patients with Chordomas: A Pilot Study. https://clinicaltrials.gov/study/NCT00713037.

[54] A Phase 2 Study of PET Imaging with 18F-FMISO and 18F-FDG for Assessment of Tumor Hypoxia in Soft Tissue Sarcoma. https://clinicaltrials.gov/study/NCT01169350.

[55] (FIPOXY) Evaluation of the Value of 18F-FMISO PET Hypoxia Imaging in Idiopathic Pulmonary Fibrosis—A Non-randomized Proof-of-concept Study Comparing Patients with Idiopathic Pulmonary Fibrosis and Healthy Subjects. https://clinicaltrials.gov/study/NCT05331729.

[56] 68Ga-RM2 PET/MRI in the Evaluation of Patients with Biochemical Recurrence of Prostate Cancer and Non-contributory CT Scans. https://clinicaltrials.gov/study/NCT02624518.

[57] A Phase II Study of 68Ga-RM2 for PET/CT of GRPr Expression in Prostate Cancer. https://clinicaltrials.gov/study/NCT02559115.

[58] 68Ga-RM2 PET/CT for Detection of Regional Nodal and Distant Metastases in Patients with Intermediate and High-Risk Prostate Cancer. https://clinicaltrials.gov/study/NCT03113617.

[59] Evaluation of the LightPath® Imaging System and the PET Tracer 68Ga-RM2 in Wide Local Excision for Breast Cancer. https://clinicaltrials.gov/study/NCT03731026.

[60] (PROSTATEP) Exploratory, Single-institution Study, Comparing 68Ga-RM2 PET/CT Versus 68Ga-PSMA-617 PET/CT in Patients Diagnosed with Intermediate Risk Prostate Cancer Candidates for Radical Prostatectomy. https://clinicaltrials.gov/study/NCT03606837.

[61] Phase II Prospective Monocentric Study on Prostate Cancer Restaging by Using PET/MR with Innovative Radiotracers. https://clinicaltrials.gov/study/NCT05806853.

[62] A Pilot Study of 68Ga-RM2 PET/MRI in the Evaluation of Patients with Estrogen Receptor-Positive Breast Cancer. https://clinicaltrials.gov/study/NCT03831711.

[63] (SABRE) 64Cu-SAR-BBN PET: A Phase 2 Study of Participants with PSMA-negative Biochemical Recurrence of Prostate Cancer. https://clinicaltrials.gov/study/NCT05407311.

[64] (BOP) Assessment of the Diagnostic Value of 64Cu-SAR-BBN PET Imaging for Men with Negative PSMA PET in Prostate Cancer. https://clinicaltrials.gov/study/NCT05613842.

[65] (COMBAT) A Phase I/IIa Theranostic Study of 64Cu-SAR-BBN and 67Cu-SAR-BBN for Identification and Treatment of GRPR-expressing Metastatic Castrate Resistant Prostate Cancer in Patients Who Are Ineligible for Therapy with 177Lu-PSMA-617. https://clinicaltrials.gov/study/NCT05633160.

[66] (MITIGATE-NeoBOMB1) A Phase I/IIa Study to Evaluate Safety, Biodistribution, Dosimetry, and Preliminary Diagnostic Performance of 68Ga-NeoBOMB1 in Patients with Advanced TKI-treated Gastrointestinal Stromal Tumor Using PET/CT. https://clinicaltrials.gov/study/NCT02931929.

[67] (NeoFIND) Phase II Study of Preliminary Diagnostic Performance of 68Ga-NeoBOMB1 in Adult Patients with Malignancies Known to Overexpress Gastrin-Releasing Peptide Receptor. https://clinicaltrials.gov/study/NCT03724253.

[68] 68Ga-NeoBOMB1 and 68Ga-PSMA R2 PET/MRI in the Evaluation of Patients with Biochemical Recurrence of Prostate Cancer. https://clinicaltrials.gov/study/NCT03698370.

[69] (NeoRay) A Phase I/IIa Open-label, Multi-center Study to Evaluate the Safety, Tolerability, Whole-body Distribution, Radiation Dosimetry and Anti-tumor Activity of 177Lu-NeoB Administered in Patients with Advanced Solid Tumors Known to Overexpress Gastrin-releasing Peptide Receptor. https://clinicaltrials.gov/study/NCT03872778.

[70] A Phase I/II, Open-label, Multi-center Trial of 177Lu-NeoB in Combination with Capecitabine in Adult Patients with Gastrin Releasing Peptide Receptor Positive, Estrogen Receptor-positive, Human Epidermal Growth Factor Receptor-2 Negative Metastatic Breast Cancer After Progression on Previous Endocrine Therapy in Combination with a CDK4/6 Inhibitor. https://clinicaltrials.gov/study/NCT06247995.

[71] A Phase Ib Dose Finding Study Assessing Safety and Activity of 177Lu-NeoB in Combination with Ribociclib and Fulvestrant in Participants with Estrogen Receptor Positive, Human Epidermal Growth Factor Receptor-2 Negative and Gastrin Releasing Peptide Receptor Positive Advanced Breast Cancer Experiencing Early Relapse From (Neo)Adjuvant Endocrine Therapy or Who Have Progressed on Endocrine Therapy in Combination with a CDK4/6 Inhibitor for Advanced Disease. https://clinicaltrials.gov/study/NCT05870579.

[72] Phase Ib Dose Finding Study Assessing Safety and Activity of 177Lu-NeoB in Combination with Radiotherapy and Temozolomide in Subjects with Newly Diagnosed Glioblastoma and as a Single Agent in Recurrent Glioblastoma. https://clinicaltrials.gov/study/NCT05739942.

[73] Assessment of the Diagnostic and Theranostic Potential of 68Ga-Bombesin PET/CT (NeoB) Imaging for Staging of ER/PR + HER2- Breast Cancer Patients with Metastatic Disease: Comparison to Conventional Imaging. https://clinicaltrials.gov/study/NCT05889728.

[74] (NEPC) A Phase I, Open-label, Multi-center Exploratory Safety and Efficacy Study with PSMA, SSTR2 and GRPR Targeted Radioligand Therapy in Metastatic Neuroendocrine Prostate Cancer. https://clinicaltrials.gov/study/NCT06379217.

[75] A Phase 2, Open-label, Proof-of-concept Study to Assess the Ability to Detect Tumors and Angiogenesis Via the Expression of ανβ3/5 Integrin Receptors by 18F-AH-111585 PET Imaging. https://clinicaltrials.gov/study/NCT00565721.

[76] A Test-retest Study to Assess Reproducibility of 18F Uptake by Solid Tumors Using PET Imaging Following Intravenous Administration of 18F-AH111585 Injection. https://clinicaltrials.gov/study/NCT00918281.

[77] (PAZPET-1) Phase 1b Exploratory Study of 18F-Fluciclatide-PET as a Marker of Angiogenic Response to Combination Therapy with the Pan-VEGF Inhibitor, Pazopanib, and Weekly Paclitaxel in Platinum Resistant Ovarian Cancer. https://clinicaltrials.gov/study/NCT01608009.

[78] (GRGDG) Diagnostic Performance and Evaluation Efficacy of Brain 68Ga-BNOTA-PRGD2 PET/CT in Pre-surgery Glioma Patients. https://clinicaltrials.gov/study/NCT01801371.

[79] (GRGDLC) Radiation Dosimetry, Plasma Pharmacokinetics, Biodistribution, Safety and Diagnostic Performance of 68Ga-BNOTA-PRGD2 in Healthy Volunteers and Lung Cancer Patients. https://clinicaltrials.gov/study/NCT01527058.

[80] Diagnosis of Metastatic Tumors on 68Ga-FAPI-RGD PET-CT and Radioligand Therapy. https://clinicaltrials.gov/study/NCT06638034.

[81] A Novel Dual-Targeting Molecular Probe TATE-RGD for the Diagnostic Integration of SSTR2 and αvβ3 Positive Tumors 68Ga-RM26-RGD PET/CT Imaging in the GRPR and αvβ3 Positive Tumor Patients. https://clinicaltrials.gov/study/NCT06632860.

[82] 68Ga-RM26-RGD PET/CT Imaging in the GRPR and αvβ3 Positive Tumor Patients. https://clinicaltrials.gov/study/NCT05549024.

[83] Therapeutic Efficiency and Response to 177Lu-AB-3PRGD2 in Patients with Integrin αVβ3 Positive Tumors. https://clinicaltrials.gov/study/NCT05013086.

[84] Phase 1/2 18F-FPPRGD2 PET/CT or PET/MRI Imaging of αvβ3 Integrins Expression as a Biomarker of Angiogenesis. https://clinicaltrials.gov/study/NCT01806675.

[85] Biodistribution and Safety of the PET Probes 18F-FPRGD2 and 18F-FPPRGD2. https://clinicaltrials.gov/study/NCT01383135.

[86] Huber M.A., Kraut N., Park J.E., Schubert R.D., Rettig W.J., Peter R.U., Garin-Chesa P. (2003). Fibroblast activation protein: Differential expression and serine protease activity in reactive stromal fibroblasts of melanocytic skin tumors. J. Investig. Dermatol..

[87] Lindner T., Loktev A., Altmann A., Giesel F., Kratochwil C., Debus J., Jager D., Mier W., Haberkorn U. (2018). Development of Quinoline-Based Theranostic Ligands for the Targeting of Fibroblast Activation Protein. J. Nucl. Med..

[88] Watabe T., Liu Y., Kaneda-Nakashima K., Shirakami Y., Lindner T., Ooe K., Toyoshima A., Nagata K., Shimosegawa E., Haberkorn U. (2020). Theranostics Targeting Fibroblast Activation Protein in the Tumor Stroma: (64)Cu- and (225)Ac-Labeled FAPI-04 in Pancreatic Cancer Xenograft Mouse Models. J. Nucl. Med..

[89] Giesel F.L., Kratochwil C., Schlittenhardt J., Dendl K., Eiber M., Staudinger F., Kessler L., Fendler W.P., Lindner T., Koerber S.A. (2021). Head-to-head intra-individual comparison of biodistribution and tumor uptake of (68)Ga-FAPI and (18)F-FDG PET/CT in cancer patients. Eur. J. Nucl. Med. Mol. Imaging.

[90] Kratochwil C., Flechsig P., Lindner T., Abderrahim L., Altmann A., Mier W., Adeberg S., Rathke H., Rohrich M., Winter H. (2019). (68)Ga-FAPI PET/CT: Tracer Uptake in 28 Different Kinds of Cancer. J. Nucl. Med..

[91] Mori Y., Novruzov E., Schmitt D., Cardinale J., Watabe T., Choyke P.L., Alavi A., Haberkorn U., Giesel F.L. (2024). Clinical applications of fibroblast activation protein inhibitor positron emission tomography (FAPI-PET). Npj Imaging.

[92] Glas A.S., Lijmer J.G., Prins M.H., Bonsel G.J., Bossuyt P.M. (2003). The diagnostic odds ratio: A single indicator of test performance. J. Clin. Epidemiol..

[93] Chang W.Y., Tseng N.C., Chen L.Y., Chang C.W., Huang Y.Y., Huang Y.T., Ou Y.C., Peng N.J. (2023). Comparison of the Detection Performance Between FAP and FDG PET/CT in Various Cancers: A Systemic Review and Meta-analysis. Clin. Nucl. Med..

[94] Zboralski D., Hoehne A., Bredenbeck A., Schumann A., Nguyen M., Schneider E., Ungewiss J., Paschke M., Haase C., von Hacht J.L. (2022). Preclinical evaluation of FAP-2286 for fibroblast activation protein targeted radionuclide imaging and therapy. Eur. J. Nucl. Med. Mol. Imaging.

[95] Jonathan McConathy M.D., Goenka A., Moy R., Menda Y., Chasen B., Khushman M., Mintz A., Zakharia Y., Sunderland J., Bowles O. (2022). 177Lu-FAP-2286 in patients with advanced or metastatic solid tumors: Initial data from a phase 1/2 study investigating safety, pharmacokinetics, dosimetry, and preliminary antitumor activity (LuMIERE). J. Nucl. Med..

[96] Pang Y., Zhao L., Meng T., Xu W., Lin Q., Wu H., Zhang J., Chen X., Sun L., Chen H. (2023). PET Imaging of Fibroblast Activation Protein in Various Types of Cancer Using (68)Ga-FAP-2286: Comparison with (18)F-FDG and (68)Ga-FAPI-46 in a Single-Center, Prospective Study. J. Nucl. Med..

[97] Lindner T., Giesel F.L., Kratochwil C., Serfling S.E. (2021). Radioligands Targeting Fibroblast Activation Protein (FAP). Cancers.

[98] Ren Y., Hao P., Dutta B., Cheow E.S., Sim K.H., Gan C.S., Lim S.K., Sze S.K. (2013). Hypoxia modulates A431 cellular pathways association to tumor radioresistance and enhanced migration revealed by comprehensive proteomic and functional studies. Mol. Cell Proteom..

[99] Reeves K.M., Song P.N., Angermeier A., Della Manna D., Li Y., Wang J., Yang E.S., Sorace A.G., Larimer B.M. (2022). (18)F-FMISO PET Imaging Identifies Hypoxia and Immunosuppressive Tumor Microenvironments and Guides Targeted Evofosfamide Therapy in Tumors Refractory to PD-1 and CTLA-4 Inhibition. Clin. Cancer Res..

[100] A Phase 2 Study of Cediranib in Combination with Olaparib in Advanced Solid Tumors. https://clinicaltrials.gov/study/NCT02498613.

[101] Ma Y., Gao F. (2024). Advances of radiolabeled GRPR ligands for PET/CT imaging of cancers. Cancer Imaging.

[102] Mansi R., Wang X., Forrer F., Waser B., Cescato R., Graham K., Borkowski S., Reubi J.C., Maecke H.R. (2011). Development of a potent DOTA-conjugated bombesin antagonist for targeting GRPr-positive tumours. Eur. J. Nucl. Med. Mol. Imaging.

[103] Haendeler M., Khawar A., Ahmadzadehfar H., Kürpig S., Meisenheimer M., Essler M., Gaertner F.C., Bundschuh R.A. (2021). Biodistribution and Radiation Dosimetric Analysis of [68Ga]Ga-RM2: A Potent GRPR Antagonist in Prostate Carcinoma Patients. Radiation.

[104] Duan H., Moradi F., Davidzon G.A., Liang T., Song H., Loening A.M., Vasanawala S., Srinivas S., Brooks J.D., Hancock S. (2024). (68)Ga-RM2 PET-MRI versus MRI alone for evaluation of patients with biochemical recurrence of prostate cancer: A single-centre, single-arm, phase 2/3 imaging trial. Lancet Oncol..

[105] Fassbender T.F., Schiller F., Zamboglou C., Drendel V., Kiefer S., Jilg C.A., Grosu A.L., Mix M. (2020). Voxel-based comparison of [(68)Ga]Ga-RM2-PET/CT and [(68)Ga]Ga-PSMA-11-PET/CT with histopathology for diagnosis of primary prostate cancer. EJNMMI Res..

[106] Wong K., Sheehan-Dare G., Nguyen A., Ho B., Liu V., Lee J., Brown L., Dear R., Chan L., Sharma S. (2022). (64)Cu-SAR-Bombesin PET-CT Imaging in the Staging of Estrogen/Progesterone Receptor Positive, HER2 Negative Metastatic Breast Cancer Patients: Safety, Dosimetry and Feasibility in a Phase I Trial. Pharmaceuticals.

[107] Kurth J., Krause B.J., Schwarzenbock S.M., Bergner C., Hakenberg O.W., Heuschkel M. (2020). First-in-human dosimetry of gastrin-releasing peptide receptor antagonist [(177)Lu]Lu-RM2: A radiopharmaceutical for the treatment of metastatic castration-resistant prostate cancer. Eur. J. Nucl. Med. Mol. Imaging.

[108] Nordquist L.T., Lengyelova E., Almaguel F., Mancini B.R., Song H., Armstrong A.J., Zurita A.J., Anderson M., Parker M., Miller R.M. (2024). COMBAT: A study of 64Cu-SAR-BBN and 67Cu-SAR-BBN for identification and treatment of GRPr-expressing metastatic castrate-resistant prostate cancer. J. Clin. Oncol..

[109] Dalm S.U., Bakker I.L., de Blois E., Doeswijk G.N., Konijnenberg M.W., Orlandi F., Barbato D., Tedesco M., Maina T., Nock B.A. (2017). 68Ga/177Lu-NeoBOMB1, a Novel Radiolabeled GRPR Antagonist for Theranostic Use in Oncology. J. Nucl. Med..

[110] Gruber L., Jimenez-Franco L.D., Decristoforo C., Uprimny C., Glatting G., Hohenberger P., Schoenberg S.O., Reindl W., Orlandi F., Mariani M. (2020). MITIGATE-NeoBOMB1, a Phase I/IIa Study to Evaluate Safety, Pharmacokinetics, and Preliminary Imaging of (68)Ga-NeoBOMB1, a Gastrin-Releasing Peptide Receptor Antagonist, in GIST Patients. J. Nucl. Med..

[111] Margadant C., Monsuur H.N., Norman J.C., Sonnenberg A. (2011). Mechanisms of integrin activation and trafficking. Curr. Opin. Cell Biol..

[112] Haubner R., Maschauer S., Prante O. (2014). PET radiopharmaceuticals for imaging integrin expression: Tracers in clinical studies and recent developments. Biomed. Res. Int..

[113] Moreno-Layseca P., Icha J., Hamidi H., Ivaska J. (2019). Integrin trafficking in cells and tissues. Nat. Cell Biol..

[114] Sleeboom J.J.F., van Tienderen G.S., Schenke-Layland K., van der Laan L.J.W., Khalil A.A., Verstegen M.M.A. (2024). The extracellular matrix as hallmark of cancer and metastasis: From biomechanics to therapeutic targets. Sci. Transl. Med..

[115] Brooks P.C., Clark R.A., Cheresh D.A. (1994). Requirement of vascular integrin alpha v beta 3 for angiogenesis. Science.

[116] Kenny L.M., Coombes R.C., Oulie I., Contractor K.B., Miller M., Spinks T.J., McParland B., Cohen P.S., Hui A.M., Palmieri C. (2008). Phase I trial of the positron-emitting Arg-Gly-Asp (RGD) peptide radioligand 18F-AH111585 in breast cancer patients. J. Nucl. Med..

[117] Zartnack F., Hennig E., Ott F., Bucherl E.S. (1983). Development and in vitro fatigue testing of a new bloodpump. Life Support. Syst..

[118] Sharma R., Valls P.O., Inglese M., Dubash S., Chen M., Gabra H., Montes A., Challapalli A., Arshad M., Tharakan G. (2020). [(18)F]Fluciclatide PET as a biomarker of response to combination therapy of pazopanib and paclitaxel in platinum-resistant/refractory ovarian cancer. Eur. J. Nucl. Med. Mol. Imaging.

[119] Wu J., Tian J., Zhang Y., Ji H., Sun J., Wang X., Sun C., Wang L., Teng Z., Lu G. (2022). (18)F-Alfatide II for the evaluation of axillary lymph nodes in breast cancer patients: Comparison with (18)F-FDG. Eur. J. Nucl. Med. Mol. Imaging.

[120] Bandyopadhyay A., Raghavan S. (2009). Defining the role of integrin alphavbeta6 in cancer. Curr. Drug Targets.

[121] Kimura R.H., Iagaru A., Guo H.H. (2023). Mini review of first-in-human integrin αvβ6 PET tracers. Front. Nucl. Med..

[122] Hausner S.H., Bold R.J., Cheuy L.Y., Chew H.K., Daly M.E., Davis R.A., Foster C.C., Kim E.J., Sutcliffe J.L. (2019). Preclinical Development and First-in-Human Imaging of the Integrin alpha(v)beta(6) with [(18)F]alpha(v)beta(6)-Binding Peptide in Metastatic Carcinoma. Clin. Cancer Res..

[123] Ganguly T., Bauer N., Davis R.A., Hausner S.H., Tang S.Y., Sutcliffe J.L. (2021). Evaluation of Copper-64-Labeled alpha(v)beta(6)-Targeting Peptides: Addition of an Albumin Binding Moiety to Improve Pharmacokinetics. Mol. Pharm..

[124] Quigley N.G., Steiger K., Hoberuck S., Czech N., Zierke M.A., Kossatz S., Pretze M., Richter F., Weichert W., Pox C. (2022). PET/CT imaging of head-and-neck and pancreatic cancer in humans by targeting the “Cancer Integrin” alphavbeta6 with Ga-68-Trivehexin. Eur. J. Nucl. Med. Mol. Imaging.

[125] Herrmann K., Schottelius M., Lapa C., Osl T., Poschenrieder A., Hanscheid H., Luckerath K., Schreder M., Bluemel C., Knott M. (2016). First-in-Human Experience of CXCR4-Directed Endoradiotherapy with 177Lu- and 90Y-Labeled Pentixather in Advanced-Stage Multiple Myeloma with Extensive Intra- and Extramedullary Disease. J. Nucl. Med..

[126] Hanscheid H., Schirbel A., Hartrampf P., Kraus S., Werner R.A., Einsele H., Wester H.J., Lassmann M., Kortum M., Buck A.K. (2022). Biokinetics and Dosimetry of (177)Lu-Pentixather. J. Nucl. Med..

[127] Dreher N., Hahner S., Fuss C.T., Schlotelburg W., Hartrampf P.E., Serfling S.E., Schirbel A., Samnick S., Higuchi T., Weich A. (2024). CXCR4-directed PET/CT with [(68) Ga]Ga-pentixafor in solid tumors-a comprehensive analysis of imaging findings and comparison with histopathology. Eur. J. Nucl. Med. Mol. Imaging.

[128] Skovgaard D., Persson M., Brandt-Larsen M., Christensen C., Madsen J., Klausen T.L., Holm S., Andersen F.L., Loft A., Berthelsen A.K. (2017). Safety, Dosimetry, and Tumor Detection Ability of (68)Ga-NOTA-AE105: First-in-Human Study of a Novel Radioligand for uPAR PET Imaging. J. Nucl. Med..

[129] Carlsen E.A., Loft M., Loft A., Berthelsen A.K., Langer S.W., Knigge U., Kjaer A. (2022). Prospective Phase II Trial of Prognostication by (68)Ga-NOTA-AE105 uPAR PET in Patients with Neuroendocrine Neoplasms: Implications for uPAR-Targeted Therapy. J. Nucl. Med..

[130] Risor L.M., Clausen M.M., Ujmajuridze Z., Farhadi M., Andersen K.F., Loft A., Friborg J., Kjaer A. (2022). Prognostic Value of Urokinase-Type Plasminogen Activator Receptor PET/CT in Head and Neck Squamous Cell Carcinomas and Comparison with (18)F-FDG PET/CT: A Single-Center Prospective Study. J. Nucl. Med..

[131] Makvandi M., Pantel A., Schwartz L., Schubert E., Xu K., Hsieh C.J., Hou C., Kim H., Weng C.C., Winters H. (2018). A PET imaging agent for evaluating PARP-1 expression in ovarian cancer. J. Clin. Investig..

[132] McDonald E.S., Doot R.K., Pantel A.R., Farwell M.D., Mach R.H., Maxwell K.N., Mankoff D.A. (2020). Positron Emission Tomography Imaging of Poly-(Adenosine Diphosphate-Ribose) Polymerase 1 Expression in Breast Cancer: A Nonrandomized Clinical Trial. JAMA Oncol..

[133] Schoder H., Franca P.D.S., Nakajima R., Burnazi E., Roberts S., Brand C., Grkovski M., Mauguen A., Dunphy M.P., Ghossein R.A. (2020). Safety and Feasibility of PARP1/2 Imaging with (18)F-PARPi in Patients with Head and Neck Cancer. Clin. Cancer Res..

[134] Michel L.S., Dyroff S., Brooks F.J., Spayd K.J., Lim S., Engle J.T., Phillips S., Tan B., Wang-Gillam A., Bognar C. (2017). PET of Poly (ADP-Ribose) Polymerase Activity in Cancer: Preclinical Assessment and First In-Human Studies. Radiology.

[135] Baum R.P., Singh A., Schuchardt C., Kulkarni H.R., Klette I., Wiessalla S., Osterkamp F., Reineke U., Smerling C. (2018). (177)Lu-3BP-227 for Neurotensin Receptor 1-Targeted Therapy of Metastatic Pancreatic Adenocarcinoma: First Clinical Results. J. Nucl. Med..

[136] Duan X., Xia L., Zhang Z., Ren Y., Pomper M.G., Rowe S.P., Li X., Li N., Zhang N., Zhu H. (2023). First-in-Human Study of the Radioligand 68Ga-N188 Targeting Nectin-4 for PET/CT Imaging of Advanced Urothelial Carcinoma. Clin. Cancer Res..

[137] Tavare R., Danton M., Giurleo J.T., Makonnen S., Hickey C., Arnold T.C., Kelly M.P., Fredriksson F., Bruestle K., Hermann A. (2022). Immuno-PET Monitoring of Lymphocytes Using the CD8-Specific Antibody REGN5054. Cancer Immunol. Res..

[138] Pandit-Taskar N., Postow M.A., Hellmann M.D., Harding J.J., Barker C.A., O’Donoghue J.A., Ziolkowska M., Ruan S., Lyashchenko S.K., Tsai F. (2020). First-in-Humans Imaging with (89)Zr-Df-IAB22M2C Anti-CD8 Minibody in Patients with Solid Malignancies: Preliminary Pharmacokinetics, Biodistribution, and Lesion Targeting. J. Nucl. Med..

[139] Kist de Ruijter L., van de Donk P.P., Hooiveld-Noeken J.S., Giesen D., Elias S.G., Lub-de Hooge M.N., Oosting S.F., Jalving M., Timens W., Brouwers A.H. (2022). Whole-body CD8(+) T cell visualization before and during cancer immunotherapy: A phase 1/2 trial. Nat. Med..

[140] Bensch F., van der Veen E.L., Lub-de Hooge M.N., Jorritsma-Smit A., Boellaard R., Kok I.C., Oosting S.F., Schroder C.P., Hiltermann T.J.N., van der Wekken A.J. (2018). (89)Zr-atezolizumab imaging as a non-invasive approach to assess clinical response to PD-L1 blockade in cancer. Nat. Med..

[141] Huisman M.C., Niemeijer A.N., Windhorst A.D., Schuit R.C., Leung D., Hayes W., Poot A., Bahce I., Radonic T., Oprea-Lager D.E. (2020). Quantification of PD-L1 Expression with (18)F-BMS-986192 PET/CT in Patients with Advanced-Stage Non-Small Cell Lung Cancer. J. Nucl. Med..

[142] Hegi-Johnson F., Rudd S.E., Wichmann C., Akhurst T., Roselt P., Trinh J., John T., Devereux L., Donnelly P.S., Hicks R. (2022). ImmunoPET: IMaging of cancer imMUNOtherapy targets with positron Emission Tomography: A phase 0/1 study characterising PD-L1 with (89)Zr-durvalumab (MEDI4736) PET/CT in stage III NSCLC patients receiving chemoradiation study protocol. BMJ Open.

[143] Zhou X., Jiang J., Yang X., Liu T., Ding J., Nimmagadda S., Pomper M.G., Zhu H., Zhao J., Yang Z. (2022). First-in-Humans Evaluation of a PD-L1-Binding Peptide PET Radiotracer in Non-Small Cell Lung Cancer Patients. J. Nucl. Med..

[144] Zhou M., Wang X., Chen B., Xiang S., Rao W., Zhang Z., Liu H., Fang J., Yin X., Deng P. (2022). Preclinical and first-in-human evaluation of (18)F-labeled D-peptide antagonist for PD-L1 status imaging with PET. Eur. J. Nucl. Med. Mol. Imaging.

[145] Juhasz C., Nahleh Z., Zitron I., Chugani D.C., Janabi M.Z., Bandyopadhyay S., Ali-Fehmi R., Mangner T.J., Chakraborty P.K., Mittal S. (2012). Tryptophan metabolism in breast cancers: Molecular imaging and immunohistochemistry studies. Nucl. Med. Biol..

[146] Zhang S., Wang X., Gao X., Chen X., Li L., Li G., Liu C., Miao Y., Wang R., Hu K. (2025). Radiopharmaceuticals and their applications in medicine. Signal Transduct. Target. Ther..

[147] Singnurkar A., Poon R., Metser U. (2024). Head-to-Head Comparison of the Diagnostic Performance of FDG PET/CT and FDG PET/MRI in Patients with Cancer: A Systematic Review and Meta-Analysis. AJR Am. J. Roentgenol..

[148] Schwenck J., Sonanini D., Cotton J.M., Rammensee H.G., la Fougere C., Zender L., Pichler B.J. (2023). Advances in PET imaging of cancer. Nat. Rev. Cancer.

[149] Herrmann K., Schwaiger M., Lewis J.S., Solomon S.B., McNeil B.J., Baumann M., Gambhir S.S., Hricak H., Weissleder R. (2020). Radiotheranostics: A roadmap for future development. Lancet Oncol..

