# Supplementary material for: Emerging PET Imaging Agents and Targeted Radioligand Therapy: A Review of Clinical Applications and Trials
Source: Tomography. 2025 Jul 28;11(8):83. doi: 10.3390/tomography11080083 (PMC12390037; doi:10.3390/tomography11080083)
Supplement: Supplementary file 1 [file tomography-11-00083-s001.zip › tomography-3711747-supplementary.pdf]

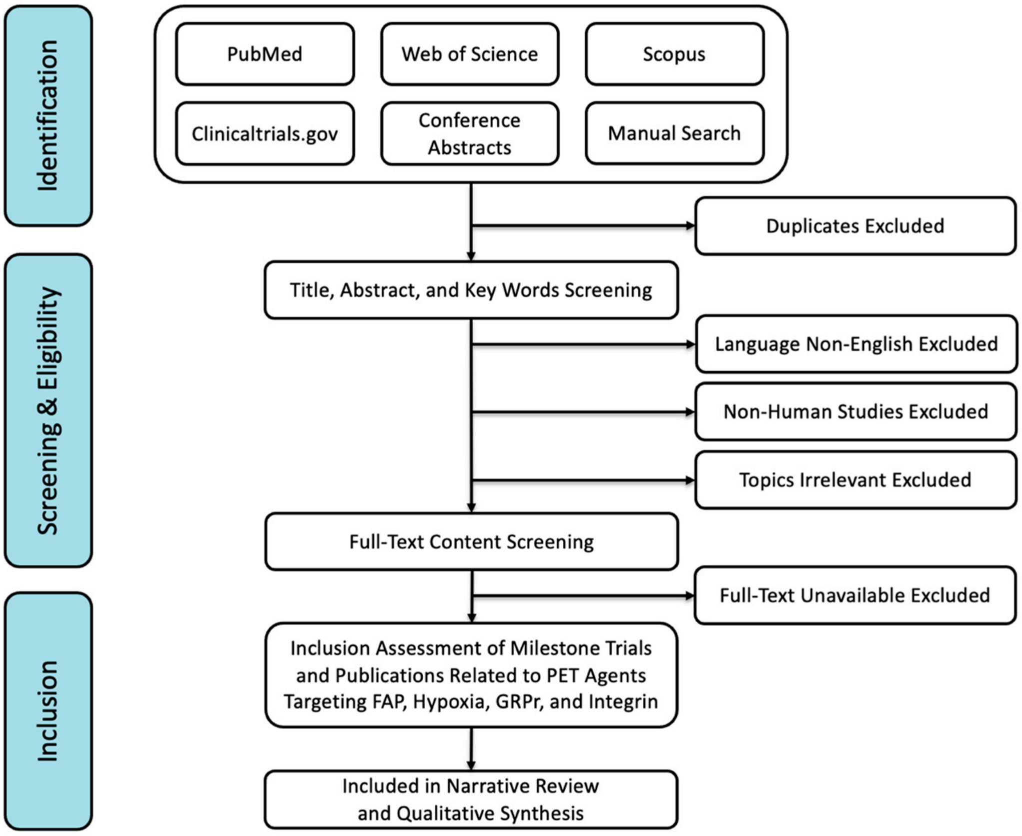

Supplementary Figure S1: The flow diagram illustrating the process of literature identification, the databases searched, and the inclusion and exclusion criteria applied during the screening and eligibility assessment of milestone publications and trials for this narrative review.
